# Mid-pregnancy poly(I:C) viral mimic disrupts placental ABC transporter expression and leads to long-term offspring motor and cognitive dysfunction

**DOI:** 10.1038/s41598-022-14248-0

**Published:** 2022-06-17

**Authors:** V. R. S. Monteiro, C. B. V. Andrade, H. R. Gomes, M. W. Reginatto, G. E. Império, K. N. Fontes, D. A. Spiess, W. S. Rangel-Junior, V. M. O. Nascimento, C. O. S. Lima, R. P. C. Sousa, F. F. Bloise, S. G. Matthews, E. Bloise, P. M. Pimentel-Coelho, T. M. Ortiga-Carvalho

**Affiliations:** 1 Instituto de Biofísica Carlos Chagas Filho, Universidade Federal do Rio de Janeiro, Rio de Janeiro, Brasil; 2 Departamento de Histologia e Embriologia, Instituto de Biologia Roberto Alcantara Gomes, Universidade Estadual Do Rio de Janeiro, Rio de Janeiro, Brasil; 3 Lunenfeld-Tanenbaum Research Institute, Mount Sinai Hospital, Toronto, Canada; 4 Department of Physiology, Faculty of Medicine, University of Toronto, Toronto, Canada; 5 Department of Obstetrics and Gynaecology, Faculty of Medicine, University of Toronto, Toronto, Canada; 6 Department of Medicine, Faculty of Medicine, University of Toronto, Toronto, Canada; 7 Departamento de Morfologia, Universidade Federal de Minas Gerais, Belo Horizonte, Brasil

**Keywords:** Infection, Inflammation, Reproductive biology, Neurodevelopmental disorders

## Abstract

Limited information is available about the effect of mid-pregnancy viral infections on the placental expression of efflux transporters and offspring behavior. We hypothesized that maternal exposure to polyinosinic-polycytidylic acid [poly(I:C)], a synthetic double-stranded RNA viral mimic, would impair placental cell turnover, the expression of selected ABC transporters and adult offspring behavior. C57BL/6 mice were administered poly(I:C) (10 mg/Kg;ip) or vehicle at gestational day (GD) 13.5 (mid-pregnancy). Dams were euthanized for blood collection 4 h after injection, fetal and placental collection at GD18.5 or allowed to deliver spontaneously at term. At GD 13.5, poly(I:C) induced an acute pro-inflammatory response characterized by an increase in maternal plasma levels of IL-6, CXCL-1 and CCL-2/MCP-1. At GD 18.5, poly(I:C) decreased cell proliferation/death in the labyrinthine and increased cell death in the junctional zones, characterizing a disruption of placental cell turnover. Abca1 and Abcg1 immunolabelling was decreased in the labyrinthine zone, whereas Abca1, Abcg1 and breast cancer resistance transporter (Bcrp) expression increased in the junctional zone. Moreover, adult offspring showed motor and cognitive impairments in the Rotarod and T-water maze tests. These results indicate that viral infection during mid-pregnancy may disrupt relevant placental efflux transporters, as well as placental cell turnover and offspring behavior in adult life.

## Introduction

The placental barrier mediates the transfer of gases, water, ions, macro and micronutrients into the fetal compartment, in addition to producing and secreting a wide range of hormones, cytokines and signaling molecules; and preventing unwanted maternal substances from reaching the fetus^1–4^. The functionally developed murine placenta comprises four distinct compartments, the decidua basalis, the junctional (Jz) and the labyrinth (Lz) zones and the chorionic plate. Of importance, the Lz and Jz are physiologically and anatomically distinct regions compared to human placenta^5–8^. The Lz is the primary site responsible for exchange of gas, nutrients and toxicants between mother and fetus, while the Jz provides structural and endocrine functions in the murine hemochorial placenta^8,9^.

Substances such as cholesterol, drugs, xenobiotics, and cytokines, are transported by a family of specialized transporters widely present in the placental barrier, the ATP-Binding Cassette (ABC) efflux transporters superfamily^4^. Among the most studied ABC transporters found in the placenta are the multidrug resistance P-glycoprotein (P-gp; encoded by *Abcb1a* and *Abcb1b* genes in rodents), the breast cancer resistance protein (Bcrp; *Abcg2*) and the lipid Abca1 (*Abca1*) and Abcg1 (*Abcg1*) transporters. Cumulative evidence from our group and others, indicate that infection and inflammation may disrupt the syncytiotrophoblast (STB) barrier efficiency, impairing the expression and function of these transporters^4,10–14^. However, there is limited information on the impact elicited by acute mid-pregnancy viral infections on the expression of ABC transporters in the different compartments of the placenta at term^12,15,16^.

It has been well established that maternal viral infection can affect placental function, which may result in pregnancy complications such as miscarriage or intrauterine growth restriction (IUGR), preterm birth and birth defects in the offspring^12,17,18^. Pregnant women are at greater risk of zika (ZIKV), cytomegalovirus (CMV) and herpes simplex viral infections, while exhibiting higher mortality rates when infected with H1N1, varicella and rubella during pregnancy—compared with the general population^17^.

Importantly, several studies have highlighted the key role of the placenta in the development of neuropsychiatric disorders in offspring. The rise of cytokine levels in response to viral infection generates a maternal inflammatory state, weakening the maternal immune system^19,20^ and increasing maternal stress levels^20,21^. Together, inflammation and stress may activate common mechanisms impairing proper fetal growth and offspring post-natal development^22^. In this context, maternal infection during gestation has been linked to a higher risk for neurodevelopmental disorders^20,22^, suggesting this insult leads to long-term changes in development^23–25^.

Polyinosinic-polycytidylic acid poly(I:C) is a synthetic analog of double-stranded RNA (dsRNA) that binds to the innate immune response toll-like receptor (TLR) 3 and mimics some aspects of a viral infection^26^, causing immune responses without the use of viral pathogens^27,28^. There is evidence that the inflammatory processes triggered by poly(I:C) may impact the expression of Bcrp/*Abcg2* and P-gp/*Abcb1a/b* placental transporters^16,29,30^ and the offspring’s behavior^31–33^. Despite this, studies are still needed to better characterize the impact of poly(I:C) on distinct placental regions and on the expression of other placental efflux transporters.

Here, we hypothesized that maternal mid-pregnancy exposure to viral analogue poly(I:C) would affect the term placenta in terms of cell turnover, ABC transporters expression, as well as would impair offspring long-term behavior. This issue is important to increase our understanding of what happens at the maternal–fetal interface during an inflammatory response that can lead to behavioral changes in the offspring.

## Results

### Mid-pregnancy poly(I:C) induces acute systemic inflammation and increases fetal/placental weight ratio at term

**Figure 1:**
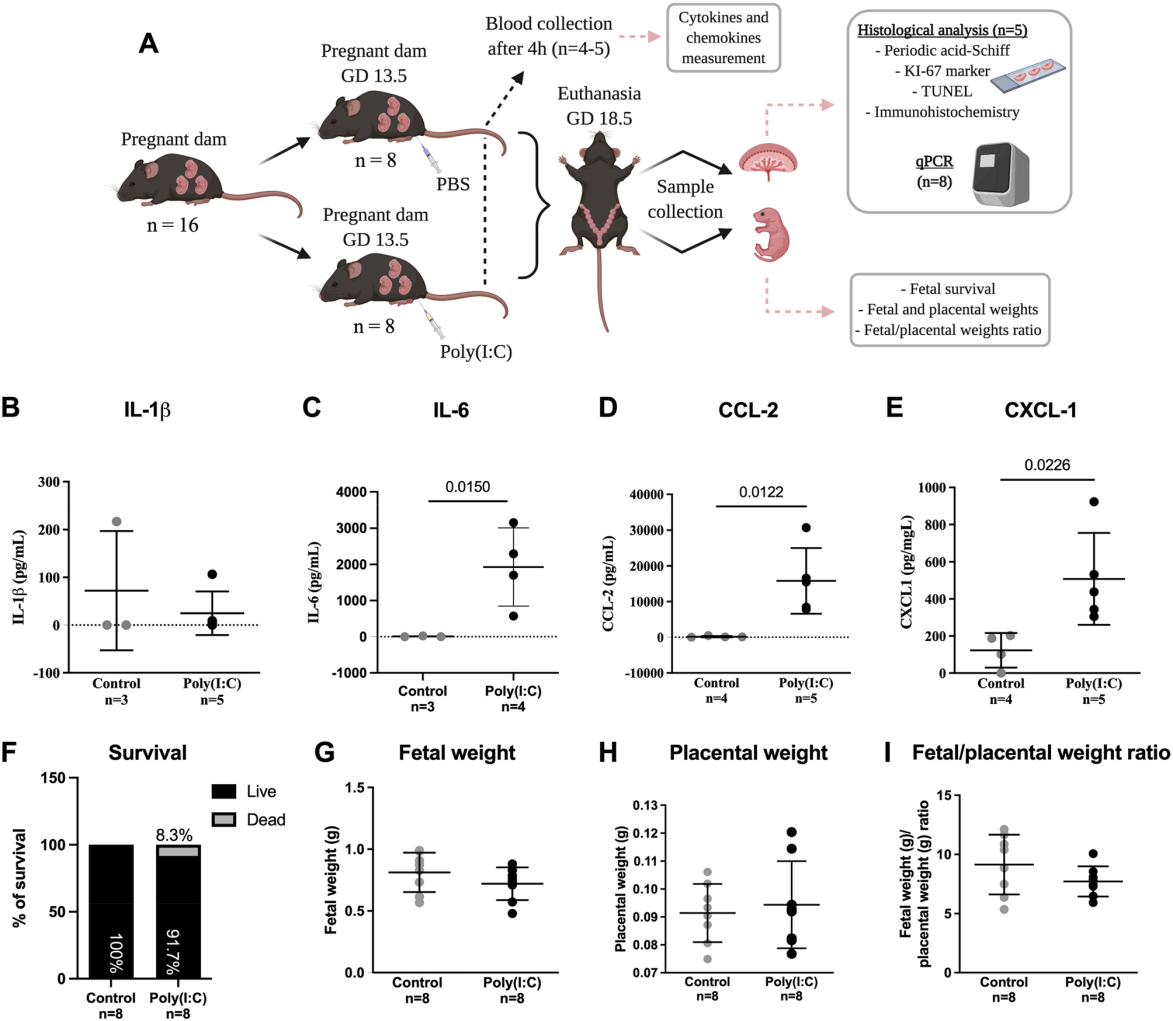
Mid-pregnancy poly(I:C) induces an acute maternal systemic inflammation and affects fetal and placental weight at term. (**A**) Experimental design (created with BioRender.com). (**B–E**) Maternal IL-1β (**B**), IL-6 (**C**), CCL-2 (**D**) and CXCL-1 (**E**) levels in maternal plasma. (**F**) Percentage of fetus survival. The N number in each group corresponds to the number of litters. (**G**–**I**) Fetal (**G**) and placental (**H**) weights, and fetal/placental ratio (**I**). The values of fetal and placental weights and their ratio correspond to the litter mean. The graphs were plotted in aligned dot plot, the gray circles represent the control group, and the black circles represent the poly(I:C) group. One outlier from control group (IL-1β), one outlier from control and poly(I:C) groups (IL-6) were removed according to Grubb’s test. Values are expressed as mean ± SD. Mann–Whitney test was performed for fetal/placental weight ratio and IL-1β. Student's t test was used for the remaining analysis.

To assess whether TLR3 activation disrupts placental efficiency and fetal biometry, the viral analogue poly(I:C) or vehicle were administered at GD 13.5 (Fig. 1A). Maternal blood was collected 4 h after the vehicle (n = 4) or poly(I:C) (n = 5) insult for cytokine/chemokine evaluation. We chose the cytokines interleukin (IL)-1β and IL-6, and chemokines (C-X-C motif) ligand 1 (CXCL-1) and C-C motif chemokine ligand 2 (CCL-2/MCP-1) based on the known effects of poly(I:C) on these inflammatory mediators^34–37^, and their involvement in neuropsychiatric disorders^38,39^. The cytokine IL-6 (Fig. 1C) and the chemokines CXCL-1 and CCL-2 (Fig. 1D,E respectively) had their levels increased in the poly(I:C) group, while the levels of IL-1β remained unchanged (Fig. 1B). These cytokines and chemokines levels remained unchanged in the fetal brains on GD 18.5 (Supplementary Fig. S1), with the exception of the chemokine CCL-2 that was increased in the female brains (Supplementary Fig. S1C).

At GD 18.5 (term), we observed a fetal death rate of 8.3% in the poly(I:C) group, with no fetal deaths detected in the control group (Fig. 1F). The fetal/placental weight ratio (F:P ratio), a surrogate marker of placental efficiency, was calculated individually for each fetus and then averaged per litter. The poly(I:C) insult was not able to impact fetal and placental weights (Fig. 1G,H) or F:P ratio (Fig. 1I).

### Mid-pregnancy poly(I:C) induces term placental remodeling in the labyrinth and junctional zones

**Figure 2:**
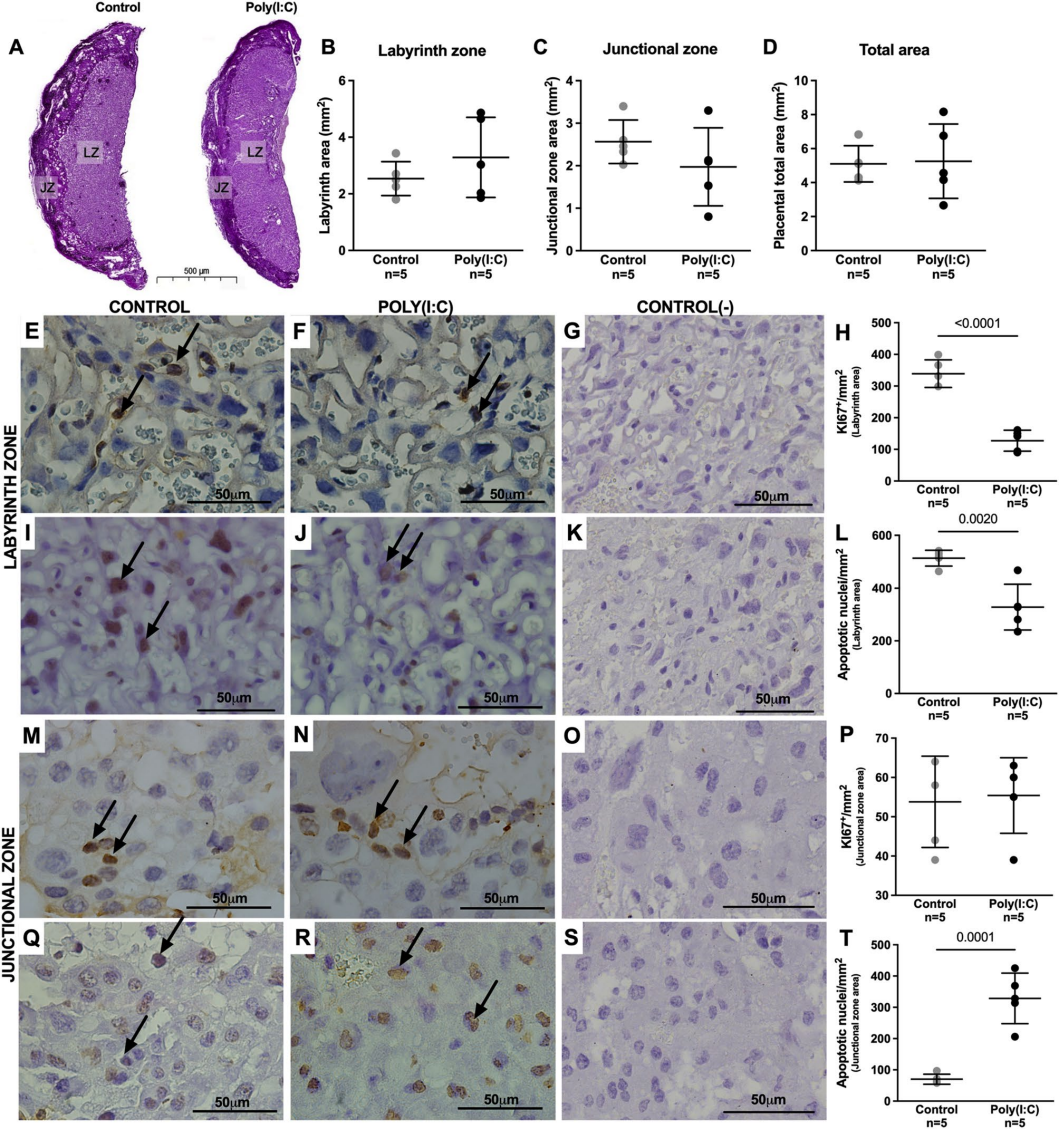
Placental remodeling after mid-pregnancy poly(I:C) exposure. (**A**) Representative images of PAS-stained at GD18.5 showing the delimitation of placental areas. (**B**–**D**) Quantification of the areas of the Lz (**B**), Jz (**C**) and total placental area (**D**). Representative images from Ki67^+^ nuclei and its quantification in Lz (**E**–**H**) and Jz (**M**–**P**). Representative images from apoptotic nuclei and its quantification in Lz (**I**–**L**) and Jz (**Q**–**T**). The graphs were plotted in aligned dot plot, the gray circles represent the control group, and the black circles represent the poly(I:C) group. The Lz’s (**G**,**K**) and Jz’s (**O**,**S**) negative controls were performed with the omission of the primary antibody. Values are expressed as mean ± SD. Student's t test was used.

We therefore investigated whether maternal exposure to poly(I:C) would cause placental remodeling, affecting these two regions differently in term placentae. There were no differences in the areas of the Lz and Jz or in the total placental area, when the poly(I:C) group was compared to the control group (Fig. 2A–D). Regarding cell proliferation, there was a lower number of Ki67-positive cells in the Lz from the poly(I:C) treated dams (Fig. 2E–H), while no difference was observed in the Jz (Fig. 2M–P). The TUNEL method showed fewer apoptotic nuclei in the Lz in the poly(I:C) group (Fig. 2I–L), whereas in the Jz an increased number of apoptotic nuclei was observed (Fig. 2Q–T).

### mRNA expression of ABC transporters is decreased in term placenta after mid-pregnancy poly(I:C) insult

**Figure 3:**
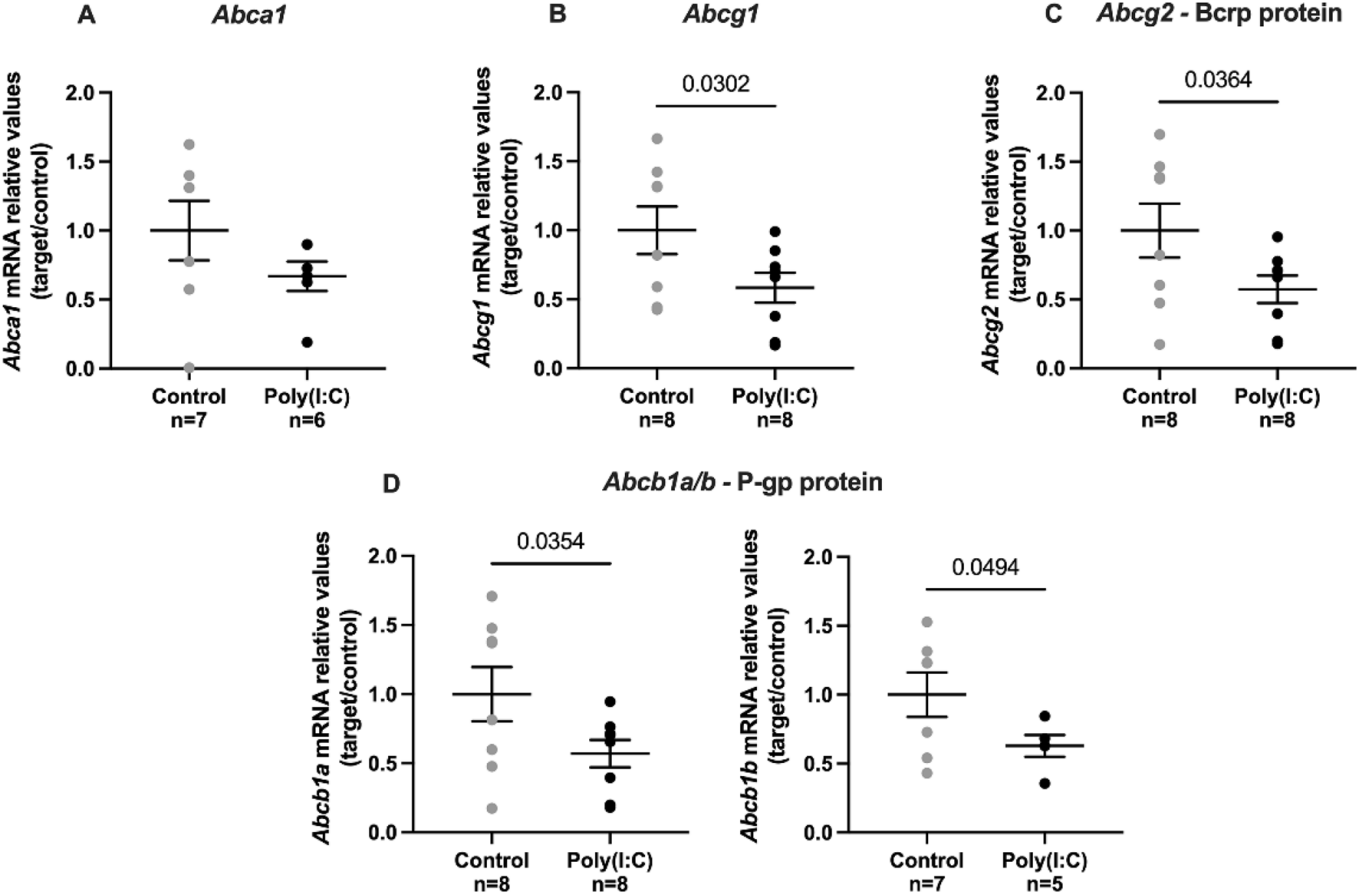
Mid-pregnancy poly(I:C) administration decreases *Abcb1a/b*, *Abcg1* and *Abcg2* mRNA expression in the placenta at GD18.5. (**A**) *Abca1*, (**B**) *Abcg1*, (**C**) *Abcg2* and (**D**) *Abcb1a/b* relative gene expression. When n < 8, samples did not pass quality checks of LinReg software and were removed from the analysis. Values are expressed as mean ± SD. Student's t-test was performed.

Total placental mRNA expression of specific ABC transporter genes at term, *Abcb1a/b*, *Abcg1* and *Abcg2* mRNA levels were decreased after mid-pregnancy poly(I:C) exposure, while placental *Abca1* remained unchanged (Fig. 3).

### Mid-pregnancy poly(I:C) changes the immunostaining intensity of ABC transporters in both the labyrinth and junctional zones in term placentas

**Figure 4:**
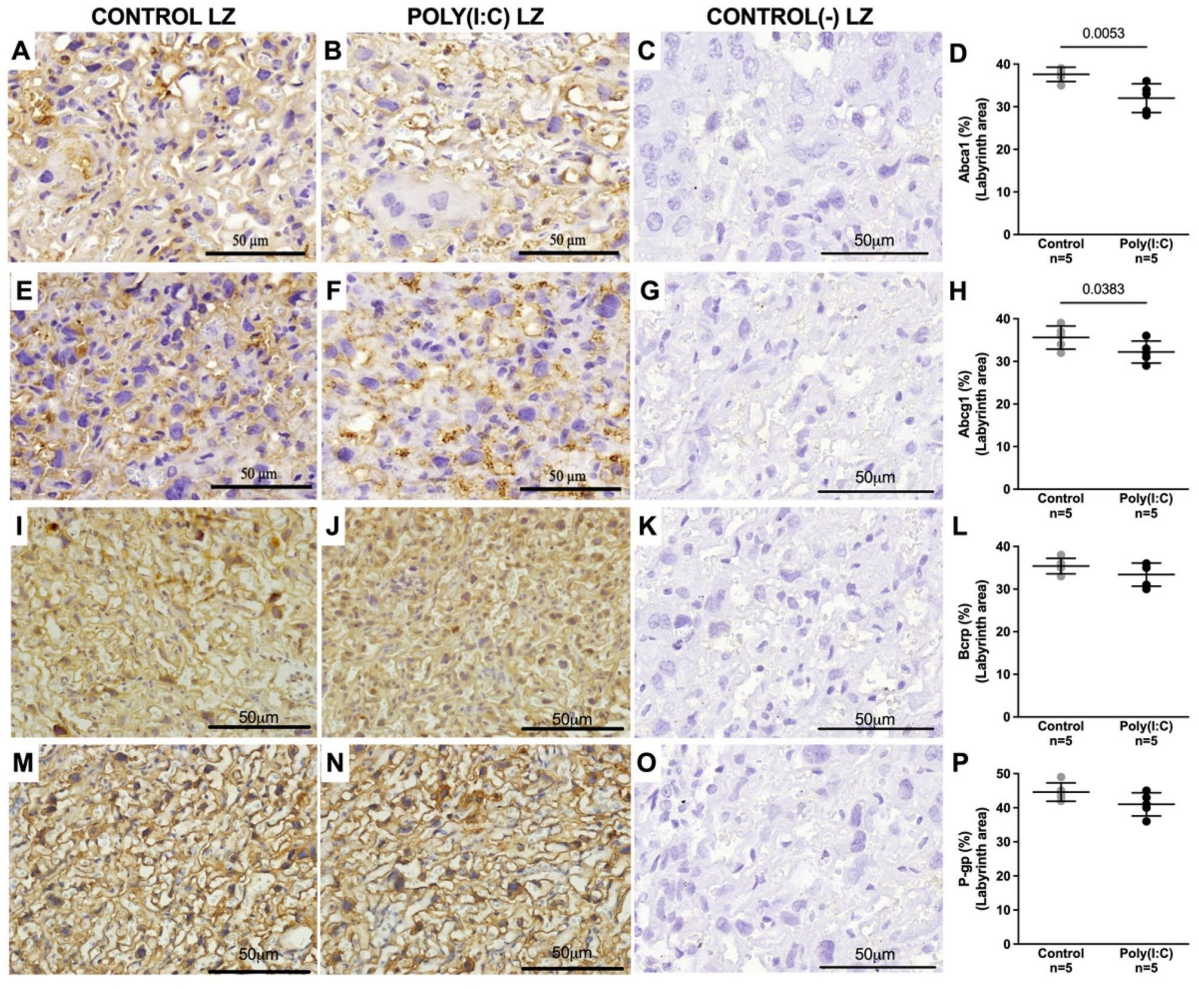
Mid-pregnancy poly(I:C) reduces Abca1 and Abcg1 immunostaining in the placental labyrinth zone (Lz) at term. Representative images of Abca1 (**A**–**D**), Abcg1 (**E**–**H**), Bcrp (**I**–**L**) and P-gp (**M**–**P**) staining and their respective quantification. Graphs were plotted in aligned dot plot, the gray circles represent the control group, and the black circles represent the poly(I:C) group. The Lz’s negative controls (**C**, G, K and **O**) were performed with the omission of the primary antibody. Values are expressed as mean ± SD. Student's t test was performed.

**Figure 5:**
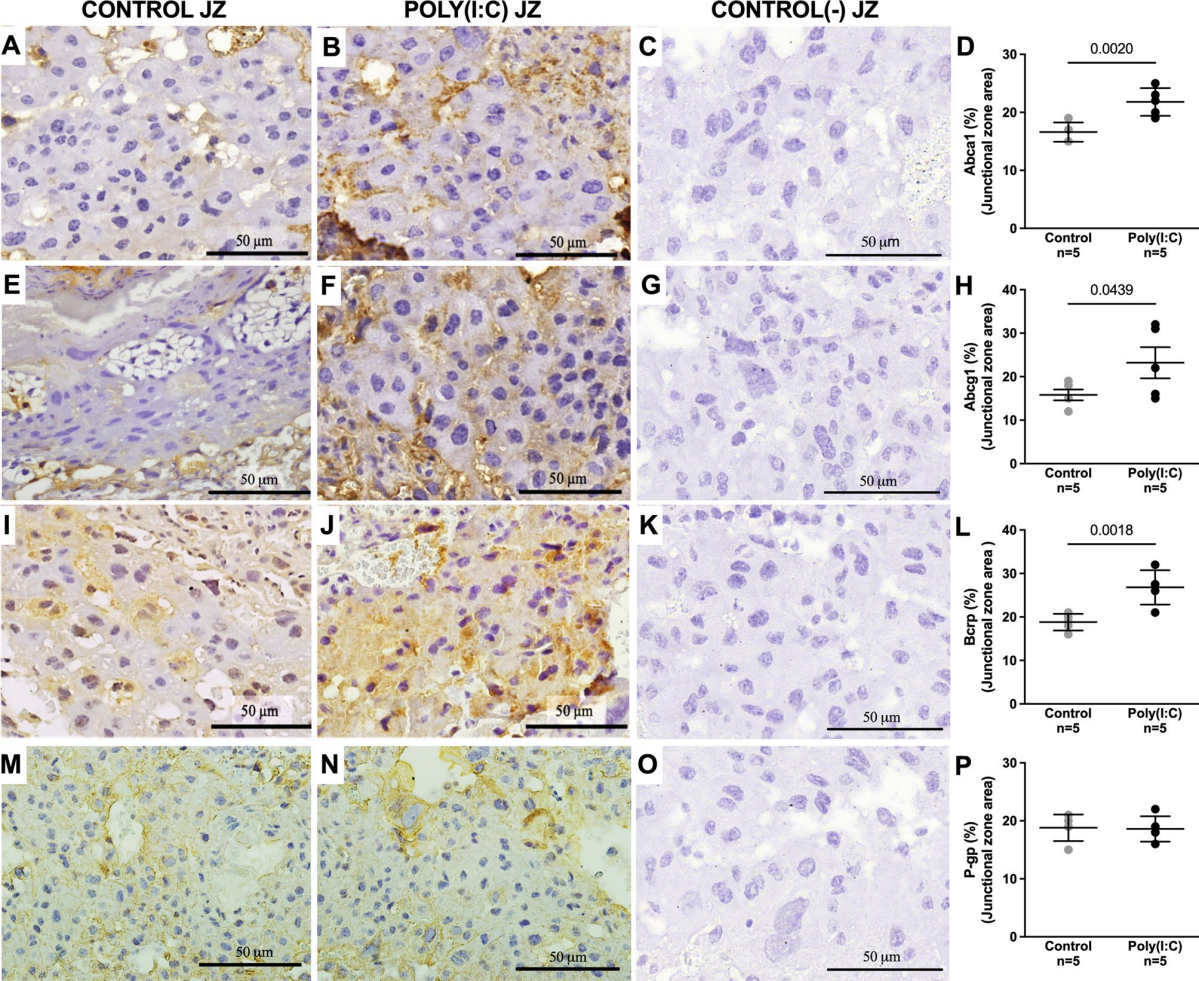
Mid-pregnancy poly(I:C) infection increases Abca1, Abcg1 and Bcrp immunostaining in the placental junctional zone (Jz) at term. Representative images of Abca1 (**A**–**D**), Abcg1 (**E**–**H**), Bcrp (**I**–**L**) and P-gp (**M**–**P**) staining and their respective quantifications. The graphs were plotted in aligned dot plot, the gray circles represent the control group, and the black circles represent the poly(I:C) group. Negative controls (**C**, G, K and **O**) were performed with the omission of the primary antibody. Values are expressed as mean ± SD. Student's t test was performed.

Abca1 (Fig. 4A–D) and Abcg1 (Fig. 4E–H) immunostaining decreased in the Lz in term placentas from the poly(I:C) group. In contrast, there was an increase in the immunostaining of these transporters in the Jz following poly(I:C) administration (Fig. 5A–H respectively). P-gp expression remained unchanged in both placental zones (Figs. 4M–P and 5M–P). The expression of Bcrp showed an increase in the Jz of the poly(I:C) group (Fig. 5I–L), whereas in the Lz it remained similar between the two groups (Fig. 4I–L).

### Motor coordination and cognitive functions in the offspring are impaired after a maternal poly(I:C) insult

**Figure 6:**
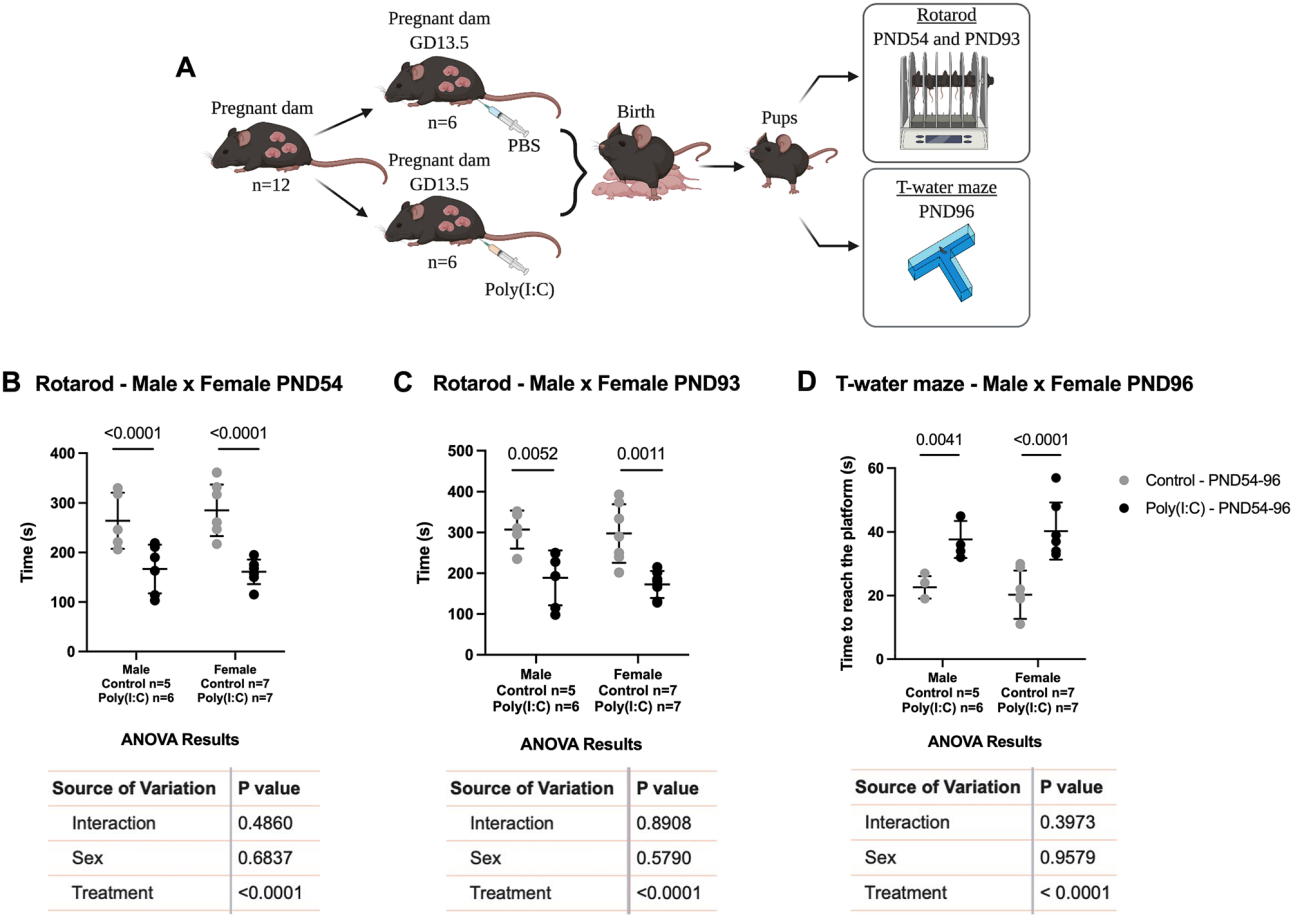
Maternal mid-pregnancy exposure to poly(I:C) induces motor coordination and cognitive deficits in both male and female offspring. (**A**) Experimental scheme (created with BioRender.com). (**B,C**) Rotarod test performed in male and female offspring at PND54 (**B**) and 93 (**C**). (**D**) T-water maze task performed at PND96. Each point represents a litter. The graphs were plotted in aligned dot plot, gray circles represent the male's and female's control groups, and black circles represent the male's and female's poly(I:C) groups. The values are plotted as individual values and they are expressed as mean ± SD. Two-way ANOVA with Šídák’s multiple comparisons test was performed.

In order to assess whether maternal mid-pregnancy exposure to poly(I:C) would cause motor and cognitive impairments in the offspring, the vehicle or the viral analogue were injected at GD13.5 and the tests were performed in the offspring at post-natal day 54 (PND54) and PND93 (Fig. 6A). Due to the fact that previous studies have identified sex differences in the impact of early adversity^21,40^, we determined whether the effects of poly(I:C) on offspring behavior were sex-specific. In the Rotarod test, both males and females born to poly(I:C) treated mothers spent less time on the rotating cylinder compared to their respective control groups at PND54 (Fig. 6B) and PND93 (Fig. 6C), indicating motor coordination deficits. Both male and female offspring from the poly(I:C) treated dams exhibited an increased latency to find the T-water maze platform, indicating deficits in spatial learning (Fig. 6D). No differences were observed in the weight of animals on test days (Supplementary Fig. S2).

## Discussion

In this manuscript, we have showed that poly(I:C) exposure in mid-pregnancy impacts fetal and placental outcomes at term (GD18.5). The impairment of motor and neurological function of the offspring at PNDs 54 and 93 caused by poly(I:C) insult during pregnancy may be caused by maternal cytokine/chemokine responses associated with placental remodeling and expression changes of specific placental ABC transporters.

Placental efficiency is defined as the relationship between the weight of the fetus and the weight of its respective placenta^41^. Previous studies have shown that poly(I:C) treatment on GD13.5 in the pregnant rats decreased placental weight, area and thickness^42^. Of all the studies involving gestational infections by different pathogens and viral mimics from our group^11–13^, poly(I:C) was not able to affect fetal weight and the fetal weight/placental weight ratio.

Few studies consider regional differences when investigating the rodent placenta. However, we previously demonstrated specific and independent responses of Lz and Jz compartments to maternal infective challenges^11–13^. In the present study, we showed that poly(I:C) affects both the Lz and Jz placental compartments, causing region specific placental remodeling. Further, ABC transporter levels in these two placental regions were affected in an opposite way following poly(I:C) treatment, reinforcing the importance of investigating these two placental compartments separately. Considering the fact that the Lz and Jz have different physiological functions, these changes could impact different aspects of fetal development.

Despite fetal weight and the fetal/placental weight ratio remaining unchanged, there was a decrease in cell proliferation in the Lz and an increase in cell death in the Jz. Considering that Lz is responsible for nutrient and gas exchanges^8,9^, a decrease in cell proliferation in this region could result in dysfunctional exchange between mother and fetus. With respect to the Jz, because it is primarily responsible for the endocrine function of the placenta^8,9^, an increase in cell death in this region may be affecting the hormonal output during pregnancy, a hypothesis that requires further investigation. Comparing with mid-pregnancy zika virus (ZIKV) infection^12^, Lz cell proliferation pattern in the poly(I:C) group was the opposite of both’s high and low dose exposed dams, whereas cell death was similar to low dose ZIKV treatment. In the Jz, both poly(I:C) and ZIKV infection did not affect cell proliferation but did increase cell death in both poly(I:C) and high dose of ZIKV. These data show that poly(I:C) viral analogue and ZIKV infection affect Lz and Jz cell turnover in a specific manner^12^.

Abca1 which is present in the apical membrane of syncytiotrophoblasts, and Abcg1, present in the basolateral membrane, primarily transport cholesterol^4^. Altered levels of these transporters can cause an imbalance in cholesterol homeostasis at the maternal–fetal interface. A decrease in Abca1 causes cholesterol to not return efficiently to the maternal compartment, accumulating in the fetal compartment, whereas a decrease in Abcg1 results in an inefficient transport of cholesterol to the fetal compartment. Pathogens can disrupt placental ABC transporters, but in a pathogen-specific manner. In the present study, poly(I:C) did not modify *Abca1* gene expression. This is similar to what we observed following maternal lipopolysaccharide (LPS, bacterial mimic) exposure^11^, but contrary to what we found in a gestational malaria model^13^. At the protein level, poly(I:C) decreased Abca1 staining in the Lz but increased staining in Jz, similar to what was found in malaria and ZIKV models^12,13^. Poly(I:C) decreased *Abcg1* gene expression in the Jz of the placenta. However, malaria infection^13^ and LPS challenge^11^ did not affect *Abcg1* mRNA levels in this region of the mouse placenta. Similar to the observed following LPS exposure^11^, poly(I:C) altered Abcg1 protein decreasing its expression in Lz and increasing it in the Jz. Given that cholesterol is essential for several development processes^43,44^, the present data would suggest that mid-pregnancy poly(I:C) treatment has the potential to impair fetal development.

P-gp (*Abcb1a/b* gene in mice) is responsible for the transport of drugs and cytokines, and Bcrp (*Abcg2* gene) transports drugs, porphyrins, and prostaglandins. Both are present in the apical membrane of the syncytiotrophoblast^4^. Previous studies have shown that acute viral infection at GD17-18, modelled through use of poly(I:C), decreases the gene expression of placental *Abcb1a*/*Abcb1b* and *Abcg2* in rats^15,16^. However, there was no effect on P-gp and Bcrp immunostaining in the placenta when the total placental area was considered^16^. Both malaria^13^ and poly(I:C) but not LPS^11^ decreased *Abcb1b* mRNA levels in the mouse placenta. In contrast to what was observed in LPS, ZIKV and malaria infections^11–13^, placental poly(I:C) exposure did not alter P-gp expression. Placental *Abcg2* mRNA decreased following poly(I:C) exposure which was similar to the response to malaria infection^13^, but contrasted with LPS exposure where no change was observed^11^. Bcrp staining did not change in the Lz following poly(I:C), whereas it decreased in the same region after LPS, malaria and ZIKV infections^11–13^. In contrast, Jz Bcrp staining increased following poly(I:C) which was similar to that seen after maternal LPS exposure^11^. The fact that P-gp and BCRP staining in the different placental regions is impacted differently in infection models highlights the importance of assessing placental regions separately. Moreover, the decreased expression of *Abcb1a/b* (P-gp) and *Abcg2* (Bcrp) in the mouse placenta following bacterial and viral challenges is consistent with findings of decreased P-gp and Bcrp expression in human placenta^10,14,29,45^. Ultimately, decreased levels of P-gp and Bcrp transporters in the placenta can result in the accumulation of substrates in the fetal compartment, which can be toxic to the fetus and impair fetal development. Therefore, although a causal relationship cannot be inferred from our experiments, it is possible that changes in the expression of efflux transporters in the placenta could also contribute to neurodevelopmental impairments, along with other factors such as the maternal immune response to viral infections. But it still needs to be studied.

Another important point that should be taken in consideration when interpreting our data is the impact of the timing of poly(I:C) exposure, as well as of the administration route, on the expression of placental ABC transporters. Even though in our model we have assessed the effects of an acute ip administration of poly(I:C) on maternal cytokine/chemokine response, we have not evaluated the acute effects of poly(I:C) on ABC transporter expression at GD13.5 or GD14.5. Previously, we have demonstrated that acute poly(I:C) exposure does not alter placental P-gp function at GD15.5, but rather impairs P-gp function in the fetal blood–brain barrier, leading to greater accumulation of the P-gp drug substrate digoxin in the fetal brain^45^. Future studies should determine whether poly(I:C) (via ip or intravaginal administration) alters the placental expression of the ABC transporters and causes placental remodeling in earlier stages of pregnancy.

The concept that maternal immune activation could affect fetal neurodevelopment was first proposed when epidemiological studies found an association between infection during pregnancy and increased rates of neuropsychiatric disorders^46^ and impairment of cognitive and affective behaviors in offspring^46–50^. It is assumed that maternal immune activation is able to influence central nervous system development by increasing local production of inflammatory cytokines^39^, in particular interleukin (IL)-6^39,51^. The role of IL-6 has been particularly well studied, and this cytokine appears to be a key mediator of neurodevelopmental outcomes in the offspring^39^. The activation of IL-6 in the placenta may trigger an inflammatory response in the fetal brain and impact the behavior of the offspring^51^. Even though there is still debate as to whether there is unidirectional transfer of cytokines across the placental barrier^52^, it has been demonstrated that maternal IL-6 can cross the placenta^53^ and in addition the placenta itself is able to produce this cytokine^39,51,53^ and impact fetal brain development. In the current study, maternal mid-pregnancy poly(I:C) exposure induced an acute rise in IL-6 levels in the maternal plasma, which may potentially have contributed to the behavioral alterations in adult offspring herein observed.

In addition, several studies have described an increase in IL-6 in maternal plasma after poly(I:C) exposure, indicating a robust systemic inflammatory response^16,36,37,54–56^. Poly(I:C) can also trigger the release of the chemokines CXCL-1 and CCL-2, possibly through the activation of TLR3^37^. TLR3 knockout mice did not show an increase in IL-6, CXCL-1 and CCL-2 in the placenta and plasma in response to poly(I:C)^55^. The release of cytokines activates the maternal immune system, which may culminate in behavioral changes. Therefore, the increase of IL-6, CXCL-1 and CCL-2 in maternal plasma could possibly underly the behavioral changes in the offspring found in the present study. With the exception of CCL-2 levels in female fetal brains (Supplementary Fig. S1C), we did not find changes in IL-6, IL-1β and CXCL-1 levels in fetal brains (Supplementary Fig. S1) probably because the measurement was made five days after the poly(I:C) insult. According to other studies, changes in cytokines levels occur acutely, with peaks at 3 h, 6 h and 24 h after the insult, and then there is a return to baseline levels^36,37,46^.

Given the sex-specificity of the effects of the early environment on offspring neurologic outcomes^21,40^, we investigated whether male and female offspring were impacted differentially following an infective challenge. Poly(I:C) led to impaired motor coordination in mice of both sexes at PND 54 and 96. Poly(I:C) also decreased cognitive abilities in both male and female offspring. Previous studies have shown that maternal immune activation can cause sensorimotor deficits^31,33,57^, as well as impairments in object recognition^32^ and social interactions in the offspring^33^. It is possible that these deficits may be related to the release of the cytokine IL-6. Animals with deletion of the IL-6 receptor gene do not exhibit behavioral changes as severe as wild type animals^53^.

In summary, we provide evidence that an acute exposure to poly(I:C) during mid-pregnancy causes placental remodeling and changes the expression of ABC transporters in both Lz and Jz at term, and that these phenotypes may potentially be related to the long-lasting behavioral dysfunction observed in the offspring. Further studies are needed to elucidate whether the change in placental transporters would be correlated with behavioral changes, and which pathways or substrates would be involved in this process.

## Material and methods

### Experimental model

All the animal experimentation in this study was approved by the Animal Care Committee of the Health Sciences Center from the Federal University of Rio de Janeiro (protocol number 036-16) and registered with the Brazilian National Council for Animal Experimentation Control. The humane animal care was in compliance with the “Principles of Laboratory Animal Care” formulated by the National Society for Medical Research and the U.S. National Academy of Sciences Guide for the Care and Use of Laboratory Animals. All experiments were performed in accordance with ARRIVE guidelines (https://arriveguidelines.org). C57Bl/6 J mice were housed in a temperature-controlled room (23 °C), under a 12/12 h light/dark cycle, and had free access to fresh food and water. Female mice aged between 8 and 10 weeks were mated overnight with male mice at a ratio of 2–3 females to each male. After 12 h, the males were removed from the cages, and if the vaginal plug was present, this day was considered as gestational day (GD) 0.5. The confirmation of pregnancy was performed by measuring the weight gain between GD0.5 and GD13.5. Weight gain of 3 g or more confirmed pregnancy. Once pregnant, dams were randomly distributed in experimental groups. For placental and fetal analysis (Fig. 1A), an intraperitoneal injection (ip) of poly(I:C) (Sigma Aldrich, Germany; Catalog Number P1530; lot #114M4028V), dissolved in 100 μL of sterile phosphate-buffered saline (PBS), was administered at a dose of 10 mg/kg at GD13.5 (n = 8). This gestational day was chosen since the murine placenta is fully formed on GD10.5. Furthermore, GD13.5 is somewhat equivalent to the first trimester of human pregnancy. Animals from the control group (n = 8) received an ip injection of the vehicle (100 μL of sterile PBS) at the same day. Maternal blood was collected through the caudal vein after 4 h of the treatment, centrifuged at 1500 G and the plasma was stored at − 20 °C. At GD 18.5 dams were euthanized with an ip injection of sodium pentobarbital at an overdose of 300 mg/kg. Each dam represents a litter (5–8 pups per litter) and the values of fetal and placental weights correspond to the litter mean per group. Placentas and fetuses were weighed, and the placentae closest to the mean weight in a litter were selected for further analysis and cut in half using umbilical cord insertion as reference^58,59^. One-half of the placental disk was frozen in liquid nitrogen for qPCR (n = 8 per group), and the other half was fixed overnight in buffered paraformaldehyde 4% (n = 5 per group; Sigma-Aldrich, Brazil) for protein immunostaining analysis. For behavioral tests, a second cohort of 6 vehicles and 6 poly(I:C) treated dams were allowed to deliver spontaneously. Here, each dam also represents a litter (5–8 pups per litter) and all newborns in the litter were evaluated per group. The resulting offspring were nursed by their dams until postnatal day (PND) 21. After weaning, they were housed under the same maternal conditions until the behavioral tests were performed at PNDs 54, 93 and 96.

### qPCR

Total placental RNA was extracted from 8 placentas from each group, using the TRIzol method according to the manufacturer’s instructions (TRIzol Reagent; Life Technologies, USA). The concentration of total RNA was assessed using NanoPhotometer (Implen, Munchen, Germany) and samples with RNA purity (260/280 absorbance) ratio ranging between 1.8 and 2.0 and with proven RNA integrity (confirmed through gel electrophoresis) were included in the study. Total RNA (1 μg) was used to synthesize cDNA using the High-Capacity cDNA Reverse Transcription Kit (Applied Biosystems, USA) according to the manufacturer’s instructions. mRNA levels of selected ABC transporters (Table 1) were evaluated by qPCR following the manufacturer’s recommendations (EVAGREEN; Solis Byodine, USA) and using the QuantStudio 3 Real-Time PCR System (Thermo Fisher, USA), with the following cycling conditions: combined initial denaturation at 50 °C (2 min) and 95 °C (10 min), followed by 40 cycles of denaturation at 95 °C (15 s), annealing at 60 °C (30 s) and extension at 72 °C (45 s). For quantification, the LinRegPCR program was used according to Ruijter et al. (2009)^60^. Tests with 95–105% efficiency were considered acceptable. Samples that did not pass in the quality checks of LinReg software (no amplification, no plateau, PCR efficiency outside 10%, excluded from mean Eff) were removed from the analysis. The reference genes were chosen according to their Cq values and variances between the groups. Gene expression was normalized to the geometric mean of reference genes, *B2m* and *Pol2a*, which exhibited stable expression levels following poly(I:C) insult. DNA contamination was ruled out using intron-spanning primers, reverse transcriptase-negative samples and melting curve analyses obtained from each qPCR reaction. All samples and standards were measured in duplicate.

**Table 1:** Primers used in the present study.

Primers	Sequence	References
*Abca1*	*F 5*′* GCAGATCAAGCATCCCAACT 3*′ *R 3*′* CCAGAGAATGTTTCATTGTCCA 5*′	Reginatto et al.^11^
*Abcb1a*	*F 5*′* GGGCATTTACTTCAAACTTGT 3*′ *R 3*′* TTTACAAGCTTCATTTCTCAA 5*′	Reginatto et al.^11^
*Abcb1b*	*F 5*′* AAGCCAGTATTCTGCCAAGCAT 3*′ *R 3*′* CTCCAGACTGCTGTTGCTGATG 5*′	Reginatto et al.^11^
*Abcg1*	*F 5*′*GGGGTCGCTCCATCATTTG 3*′ *R 3*′*TTCCCCGGTACACACATTGTC 5*′	*
*Abcg2*	*F 5*′* TGCCAGGCGCTCATTTAAAAACTTGC 3*′ *R 3*′* GCATTCCAGCGGCATCATATTTCAGA 5*′	Reginatto et al.^11^ and Fontes et al.^13^
*B2m*	*F 5*′*TTCTGGTGCTTGTCTCACTGA 3*′ *R 3*′*CAGTATGTTCGGCTTCCCATTC 5*′	*
*Pol2a*	*F 5*′*TCTGCCAAGAATGTGACGCT 3*′ *R 3*′*CCAAGCGGCAAAGAATGTCC 5*′	*

*Gene specific primers were designed with primer-BLAST (http://www.ncbi.nlm.gov/tools/primer-blast).

### Histological, immunohistochemical and TUNEL analysis

Five fixed placentas from 5 pregnancies of each group were processed following a protocol with increasing concentrations of ethanol (Isofar, Brazil), diaphanization (or clarification) with xylol (Isofar, Brazil) and inclusion in paraffin (Easypath, Brazil), followed by tissue sectioning in 5 μm thickness using a Rotatory Microtome CUT 5062 (Slee Medical GmbH, Germany). Slides were then subjected to Periodic Acid-Schiff (PAS) staining, immunohistochemistry or terminal deoxynucleotidyl transferase dUTP nick end labeling (TUNEL) assay, as described below.

For PAS staining, following diaphanization with three xylol baths, and hydration with decreasing concentrations of ethanol (100%, 90%, and 70%), placental sections were oxidized with 0.5% periodic acid (Sigma-Aldrich, USA) for 20 min, washed in flowing water and incubated with Schiff’s reagent (Merck Millipore, Germany) for 10 min at room temperature.

For immunohistochemical analysis, after deparaffinization and rehydration, sections were exposed to hydrogen peroxide (3%) for 30 min and washed with PBS containing 0.2% Tween. Antigen recovery was achieved by immersing the slides in Tris–EDTA buffer (pH 9.0) followed by immersion in sodium citrate buffer (pH 6.0) as previously described^13^. The slides were then incubated in bovine serum albumin (3%) in PBS for 1 h to block non-specific antibody binding sites. Slides were then incubated overnight at 4 °C with one of the following primary antibodies: anti-Ki67 (1:100—[M3064]; Spring Bioscience, USA), anti-P-gp (1:500—[Sc55510]; Santa Cruz Biotechnology, USA), anti-Bcrp (1:100—[MAB4146]; Merck Millipore), anti-Abca1 (1:200—[ab18180]; Abcam Plc, UK) or anti-Abcg1 (1:100—[PA5-13462]; Thermo Fisher, USA). The next day, the slides were incubated with a biotin-conjugated secondary antibody (SPD-060; Spring Bioscience, USA) for 1 h at room temperature. Subsequently, slides were incubated with streptavidin (SPD-060 – Spring Bioscience, USA) for 1 h, and with 3,3-diamino-benzidine (DAB) (SPD-060 – Spring Bioscience, USA) for 2 min.

The TUNEL method was used for the detection of apoptotic nuclei, using the ApopTag® In Situ Peroxidase Detection Kit (Merck Millipore, USA), according to the manufacturer’s recommendations.

After PAS staining, immunohistochemistry or TUNEL staining, the sections were counterstained with hematoxylin, dehydrated in increasing concentrations of ethanol (70%, 90%, and 100%), immersed in 3 baths of xylol and mounted with coverslips using Entellan (Merck, Germany). Image acquisition was performed using a high-resolution Olympus DP72 (Olympus Corporation, Japan) camera coupled to an Olympus BX53 light microscope (Olympus Corporation, Japan). Fifteen digital images from different random fields were captured per tissue fragment of each placental zone (Lz and Jz) in 5 control mice and 5 poly(I:C)-treated mice, with a total of 300 digital images for each analysis. For the analysis of PAS-stained sections, the area of each region was measured using the free-drawing tool of the Image J software (National Institutes of Health, USA). Quantification of Ki-67 and TUNEL immunostained nuclei was performed using the STEPanizer software^61^. Quantification of the area stained by P-gp, BCRP, Abca1 and Abcg1 staining was performed with the mask tool present in the Image Pro Plus 5.0 software. The percentage of viable tissue area was considered upon exclusion of negative spaces. All negative controls were performed with the omission of the primary antibody. For all analysis, the examiner was blinded to group allocation.

### Measurement of plasma cytokine and chemokine levels

Blood samples from the control (n = 4) and poly (I:C) (n = 5) treated dams were collected 4 h after the intraperitoneal injection of poly(I:C) or vehicle to confirm the expected change of cytokines and chemokines. Maternal plasma IL1-β, IL-6, monocyte chemoattractant protein-1 (MCP-1/CCL2) and chemokine (C-X-C motif) ligand 1 (KC-CXCL1) concentrations were measured using the commercially available MILLIPLEX-MAP Mouse Cytokine/Chemokine Magnetic Bead Panel – Immunology Multiplex Assays (Merck Millipore, USA), according to manufacturer’s protocol recommendations. Fluorescence intensity was detected using a MAGPIX® System (Merck Millipore, Germany). Minimum detectable concentration (MiDC): IL1-β = 12.7 pg/mL, IL-6 = 2.1 pg/mL, CCL2 = 4.1 pg/mL and CXCL1 = 2.0 pg/mL. Values below the MiDC were considered as zero.

### Offspring’s behavioral analysis

The rotarod test was used to evaluate motor coordination, while the T-water maze was employed for the evaluation of cognitive function (spatial learning). All behavioral tests were carried out in a silent and dimly lit experimental room in the afternoon, and the order of mice was randomized. The examiner was blinded to treatment allocation and the results were analyzed considering the mean of each litter.

#### Rotarod

The rotarod performance test was performed at PND54 and 93. The animals were placed in a neutral position and the rod was set to accelerate from 3 to 37 rpm in 5 min. Mice were subjected to three trials per session, with an interval of 10 min between each trial. The latency to fall was recorded automatically in each trial. The longest time spent on the rotarod was chosen for each animal.

#### T-water maze

The t-water maze task was performed at PND96. In this task, the ability of the mouse to remember the spatial location of a submerged platform was evaluated. The T-maze apparatus (length of stem, 60 cm; length of arms, 45 cm; width, 19 cm; height of walls, 20 cm) was made of clear fiberglass and filled with water (23 ± 1 °C) at a height of 15 cm. An escape platform (17.5 × 14 cm) was placed at the end of the target arm and was submerged 1 cm below the surface. The position of the platform was chosen randomly for each animal before testing. In this test, which allows the evaluation of left–right spatial learning, the mice were placed in the stem of the T-maze and swam freely until they found the submerged platform (located either in the right or in the left arm of the T-maze apparatus) and escaped to it. If the animals did not find the platform within 60 s, they were gently guided onto it. After reaching the platform, the mice remained on it for 20 s. The time to reach the platform was recorded in a training trial (trial 1) and in 3 testing trials (trials 2–4). The average of the three testing trials per litter was used for statistical analysis.

### Statistical analysis

To perform the statistical analysis, we used Graphpad Prism 9 (GraphPad Software, Inc., USA). All results were expressed as mean ± standard deviation (SD). Grubb’s test was used to identify outliers (alpha = 0.05%, check the most extreme value at either side). Outliers were removed from the analysis. The D’Agostino & Pearson omnibus and Shapiro–Wilk normality tests were used. When the groups did not pass normality test, a non-parametric test was used. Comparisons between two groups were performed using the unpaired Student’s t test, or Mann–Whitney U test (IL-1β levels in maternal plasma). Two-way ANOVA with Šídák’s multiple comparisons test was performed for the behavioral analysis, considering the factors sex and treatment. Statistical differences were considered significant when *p* < 0.05.

## Supplementary Information

Supplementary Information.

## Data Availability

The datasets generated and analyzed during the current study are available in the Mendeley data repository: https://data.mendeley.com/datasets/c98p29m3g9/1.
