# Supplementary material for: Mid-pregnancy poly(I:C) viral mimic disrupts placental ABC transporter expression and leads to long-term offspring motor and cognitive dysfunction
Source: Sci Rep. 2022 Jun 17;12:10262. doi: 10.1038/s41598-022-14248-0 (PMC9205917; doi:10.1038/s41598-022-14248-0)
Supplement: Supplementary file 1 — Supplementary Information. [file 41598_2022_14248_MOESM1_ESM.docx]

Supplementary Information for

**Mid-pregnancy poly(I:C) viral mimic disrupts placental ABC transporter expression and leads to long-term offspring motor and cognitive dysfunction**

Monteiro VRS^1^, Andrade CBV^2^, Gomes HR^1^, Reginatto MW^1^, Império GE^3^, Fontes KN^1^, Spiess DA^1^, Rangel-Junior WS^1^, Nascimento VMO^1^, Lima COS^1^, Sousa RPC^1^, Bloise FF^1^, Matthews SG^3,4,5,6^, Bloise E^7^, Pimentel-Coelho PM^1^, Ortiga-Carvalho TM*^1^.

Instituto de Biofísica Carlos Chagas Filho^1^, Universidade Federal do Rio de Janeiro, Rio de Janeiro, Brasil. Departamento de Histologia e Embriologia^2^, Instituto de Biologia Roberto Alcantara Gomes, Universidade Estadual do Rio de Janeiro, Rio de Janeiro, Brasil. Lunenfeld-Tanenbaum Research Institute^3^, Mount Sinai Hospital, Toronto, Canada. Department of Physiology^4^, Faculty of Medicine, University of Toronto, Toronto, Canada. Department of Obstetrics & Gynaecology^5^, Faculty of Medicine, University of Toronto, Toronto, Canada. Department of Medicine^6^, Faculty of Medicine, University of Toronto, Toronto, Canada. Departamento de Morfologia^7^, Universidade Federal de Minas Gerais, Belo Horizonte, Brazil.

Correspondent author: Tania Maria Ruffoni Ortiga

Instituto de Biofísica Carlos Chagas Filho, Universidade Federal do Rio de Janeiro, Rio de Janeiro, Brasil.

Tel: +55 21 39386535

E-mail: [taniaort@biof.ufrj.br](mailto:taniaort@biof.ufrj.br)

***Measurement of Cytokine and Chemokine levels in fetal brain***

Two fetal brains (1 male and 1 female) from 7 litters obtained from gestational day 18.5 were homogenized in extraction buffer (50mM Tris, 150mM NaCl, 1X Triton, 0.1% SDS, 5mM EDTA, 5mM NaF, 50mM sodium pyrophosphate, 1mM sodium orthovanadate, pH 7.4) containing a cocktail of complete protease inhibitors (Roche Applied Science, Germany) in TissueLyser LT (Qiagen, Germany). The protein concentration of each sample was analyzed using the Pierce^TM^ BCA Protein Assay Kit (Thermo Scientific, USA), according to the manufacturer's instructions. IL1-β, IL-6, monocyte chemoattractant protein-1 (MCP-1/CCL2) and chemokine (C-X-C motif) ligand 1 (KC/CXCL1) concentrations were measured using the commercially available MILLIPLEX-MAP Mouse Cytokine/Chemokine Magnetic Bead Panel – Immunology Multiplex Assays (Merck Millipore, USA), according to manufacturer’s protocol recommendations. Fluorescence intensity was detected using a MAGPIX® System (Merck Millipore, Germany). Minimum detectable concentration (MiDC): IL1-β = 12.7 pg/mL, IL-6 = 2.1 pg/mL, CCL2 = 4,1 pg/mL and CXCL1 = 2.0 pg/mL. Values below the MiDC were considered as zero. The ratio between the cytokine levels and the total protein levels was calculated for each sample. The results were analyzed considering the mean of each litter or the values of each animal separately.

***Statistical analysis***

To perform the statistical analysis, we used Graphpad Prism 9 (GraphPad Software, Inc., USA). All results were expressed as mean ± standard deviation (SD). Grubb’s test was used to identify outliers (alpha = 0.05%). Outliers were removed from the analysis. The D’Agostino & Pearson omnibus and Shapiro-Wilk normality tests were used. Comparisons between two groups were performed using the unpaired Student’s t test. Statistical differences were considered significant when p<0.05.


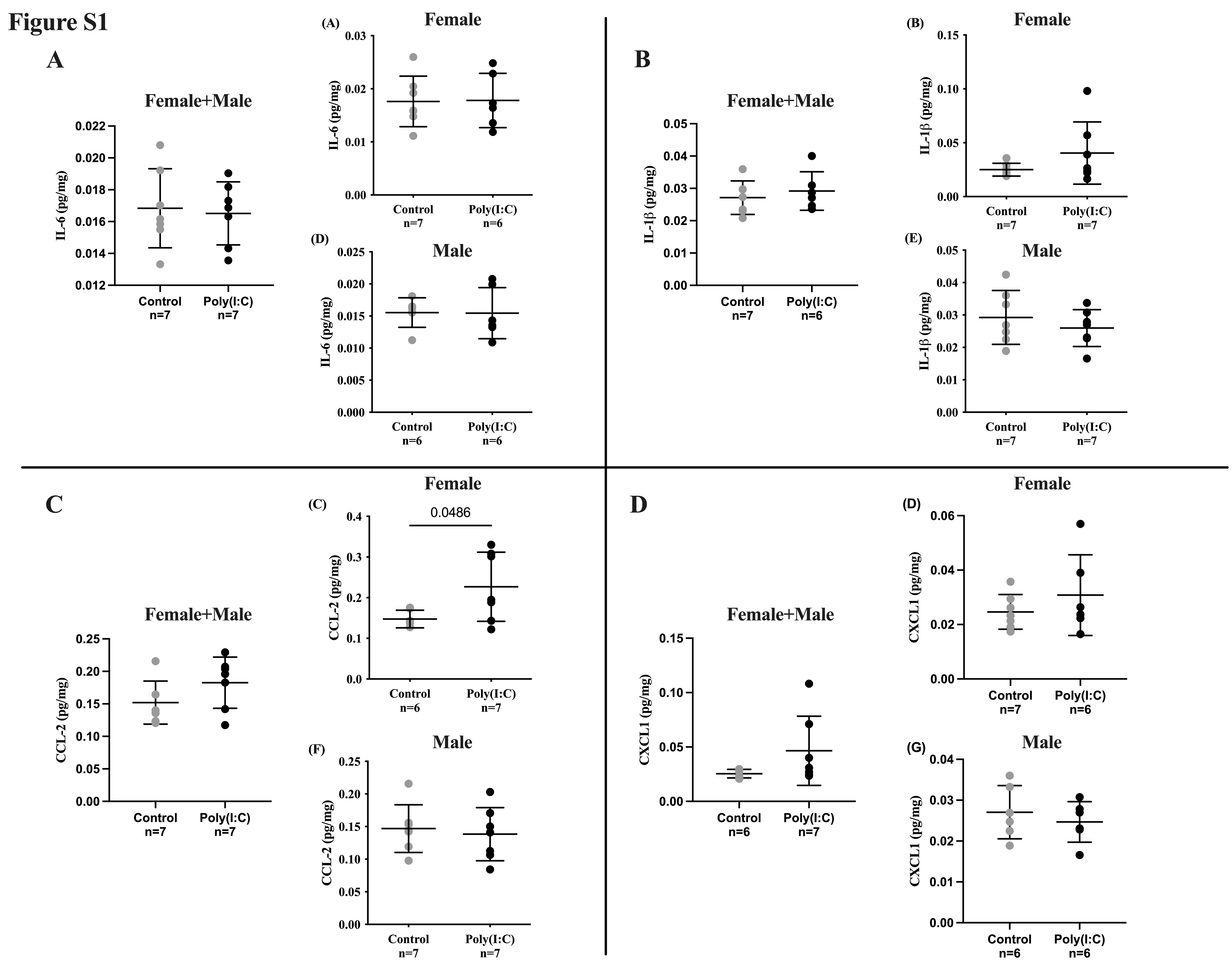


**Figure S1: Cytokines and chemokines in fetal brains.** (A) IL-6, (B) IL-1β, (C) CCL-2 and (D) CXCL-1. The N number in each group corresponds to the number of litters (1 male and 1 female). The graphs were plotted in aligned dot plot, the gray circles represent the control group, and the black circles represent the poly(I:C) group. When n<7, there were samples identified as outliers that were removed according to the Grubb’s test. Values are expressed as mean ± SD. Student's t test was performed.


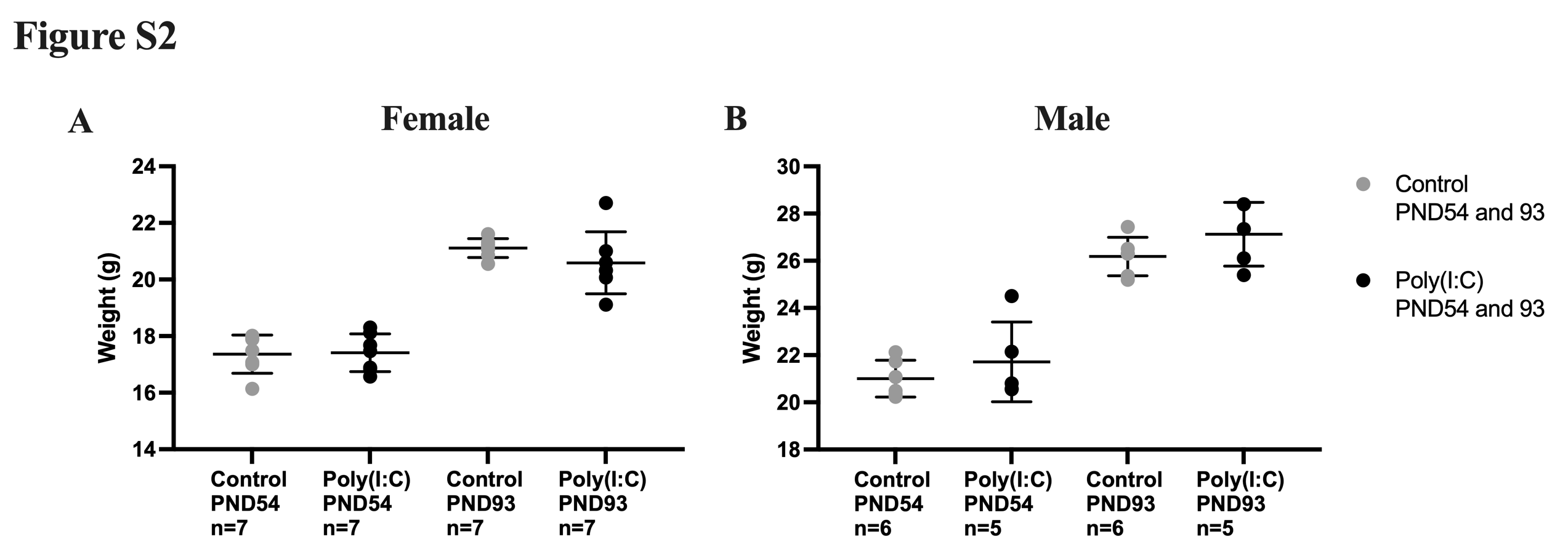


**Figure S2: Offspring weights on test days.** Female (A) and male (B) weights on PND54 and 93. The N number in each group corresponds to the number of litters. The graphs were plotted in aligned dot plot, the gray circles represent the control group, and the black circles represent the poly(I:C) group. Values are expressed as mean ± SD. Student's t test was performed.
